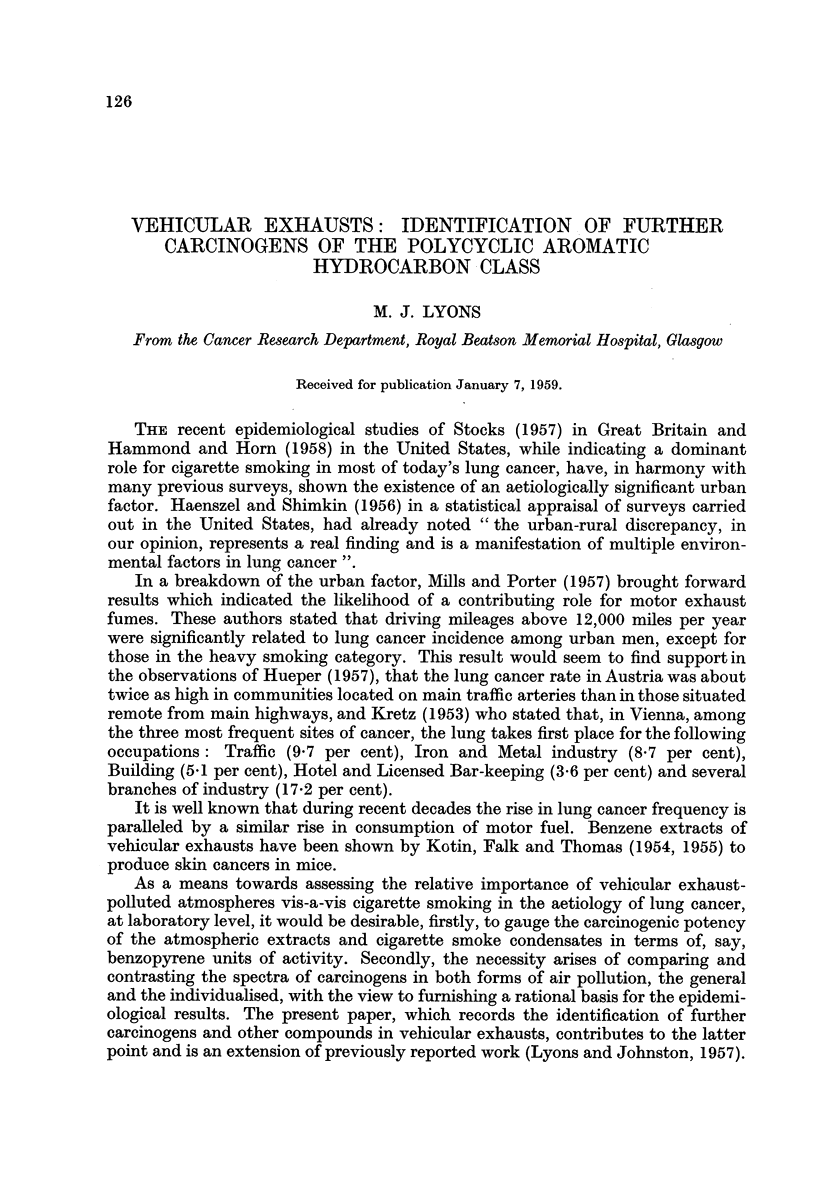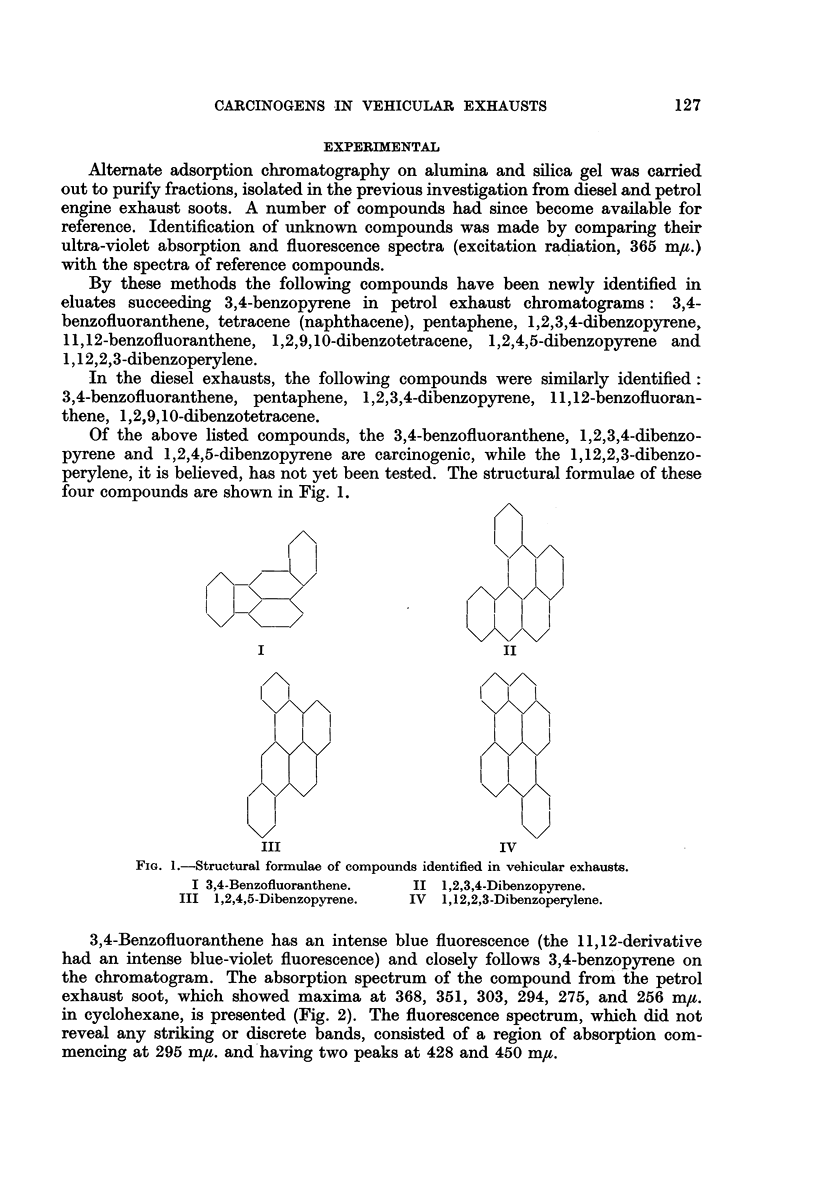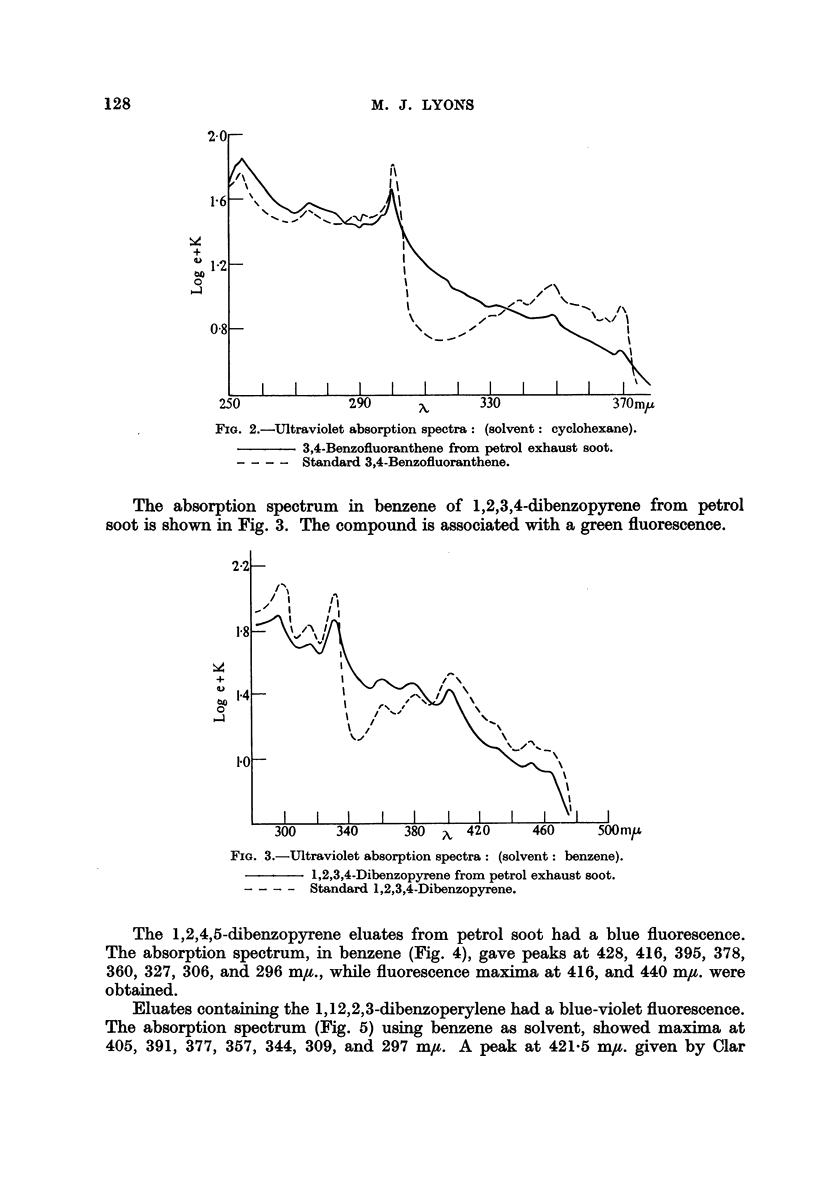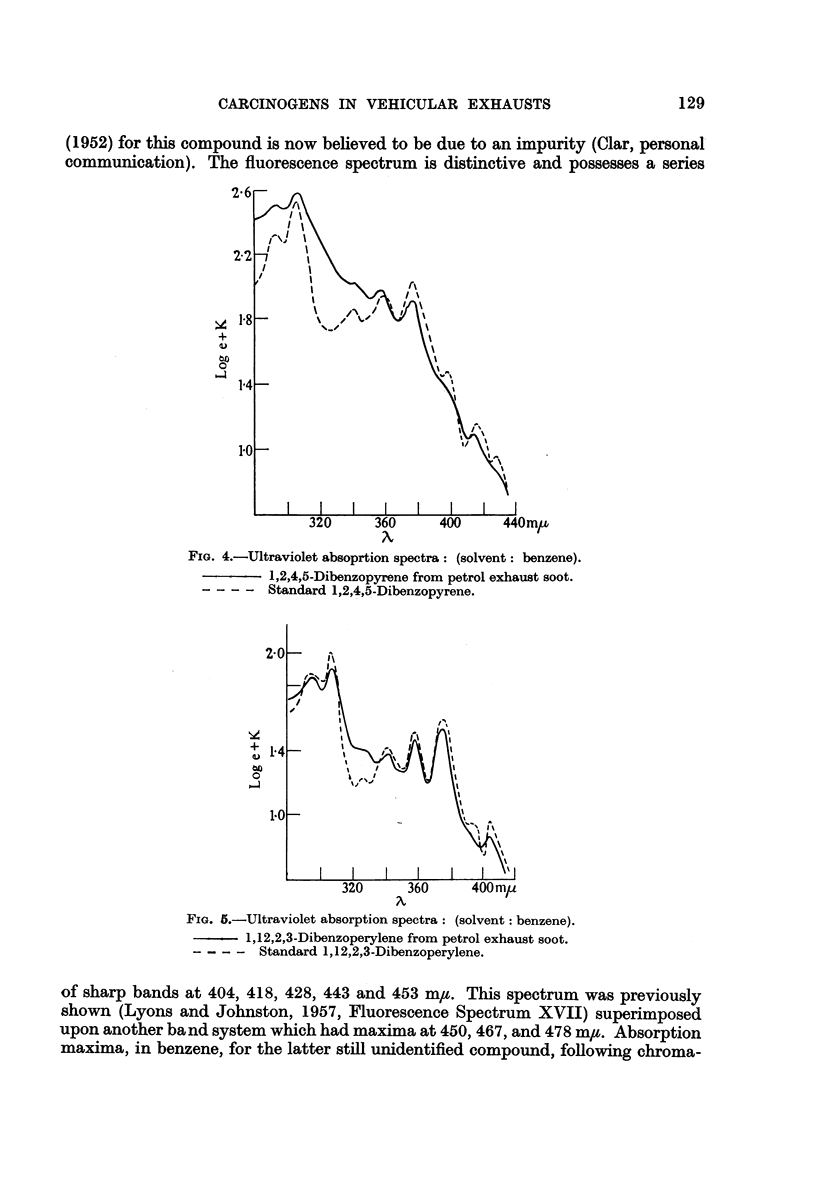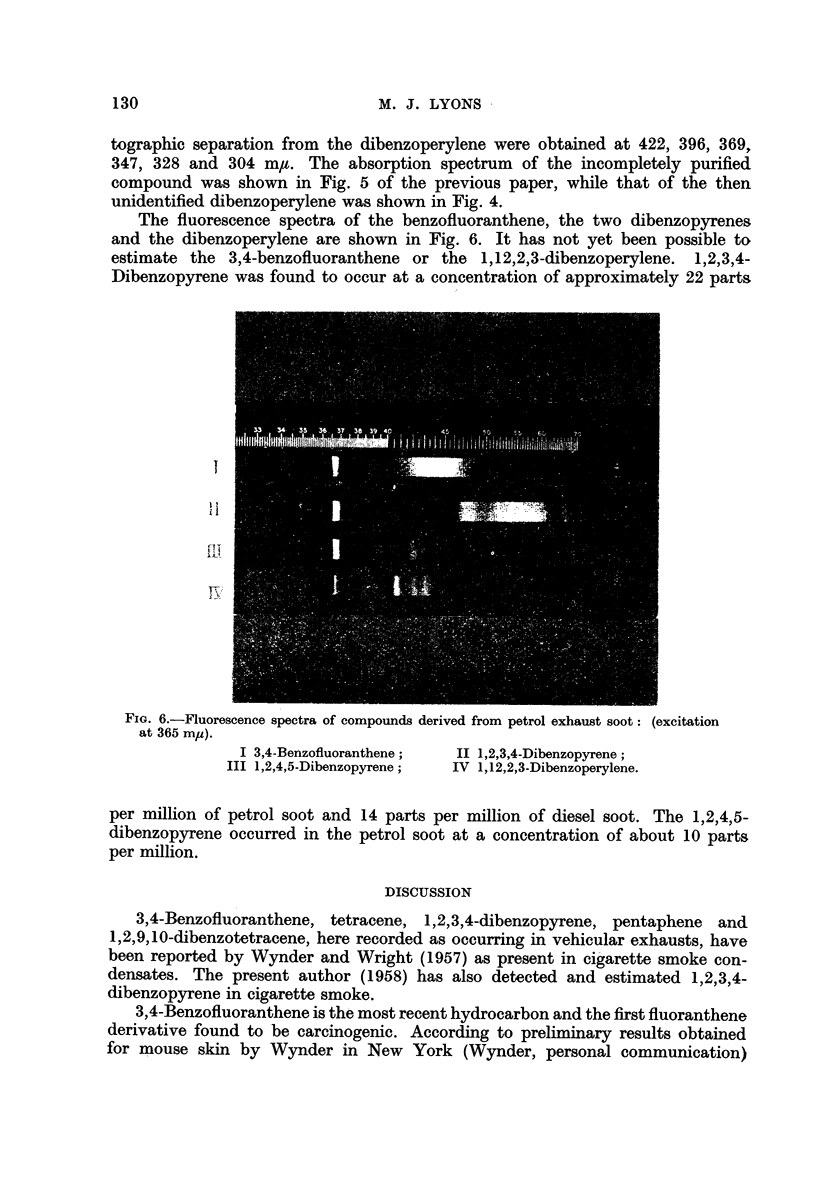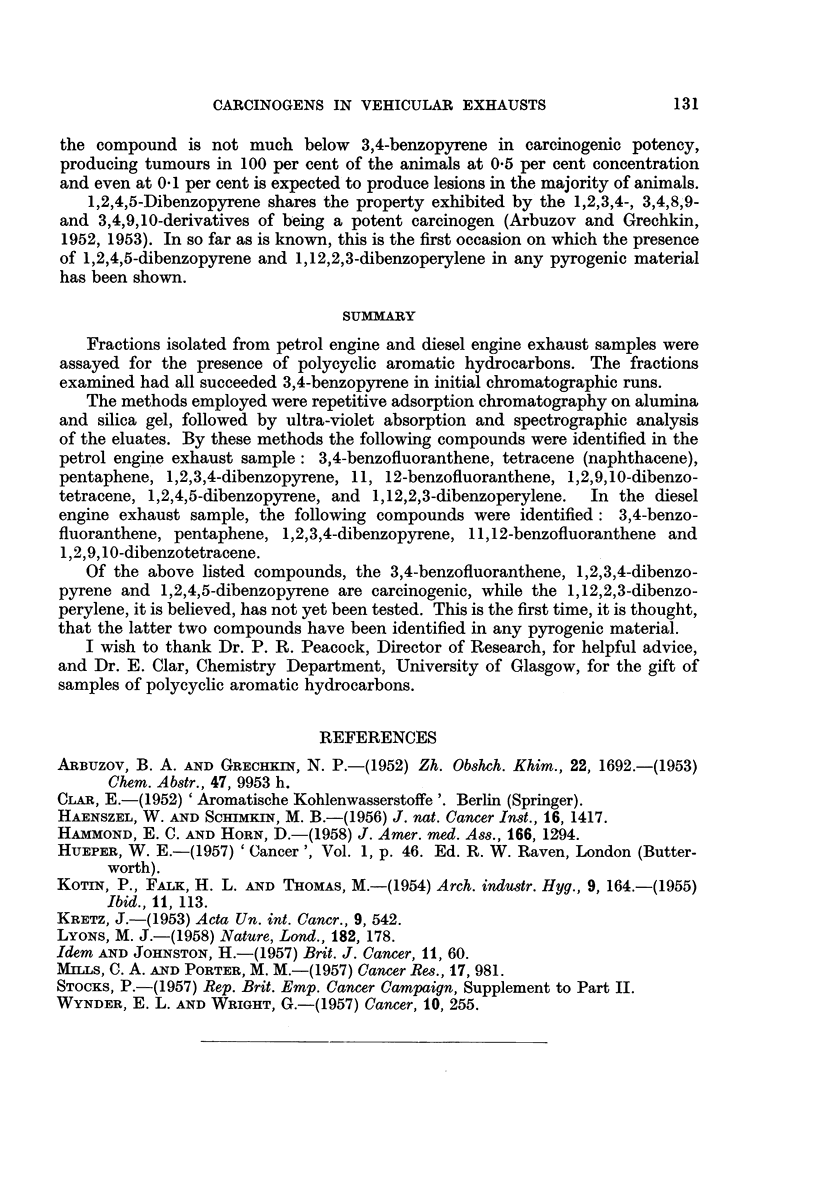# Vehicular Exhausts: Identification of further Carcinogens of the Polycyclic Aromatic Hydrocarbon Class

**DOI:** 10.1038/bjc.1959.18

**Published:** 1959-03

**Authors:** M. J. Lyons

## Abstract

**Images:**


### VEHICULAR EXHAUSTS: IDENTIFICATION OF FURTHER CARCINOGENS OF THE POLYCYCLIC AROMATIC HYDROCARBON CLASS

M. J. LYONS

*From the Cancer Research Department, Royal Beatson Memorial Hospital, Glasgow*

Received for publication January 7, 1959.

THE recent epidemiological studies of Stocks (1957) in Great Britain and Hammond and Horn (1958) in the United States, while indicating a dominant role for cigarette smoking in most of today's lung cancer, have, in harmony with many previous surveys, shown the existence of an aetiologically significant urban factor. Haenszel and Shimkin (1956) in a statistical appraisal of surveys carried out in the United States, had already noted " the urban-rural discrepancy, in our opinion, represents a real finding and is a manifestation of multiple environmental factors in lung cancer ".

In a breakdown of the urban factor, Mills and Porter (1957) brought forward results which indicated the likelihood of a contributing role for motor exhaust fumes. These authors stated that driving mileages above 12,000 miles per year were significantly related to lung cancer incidence among urban men, except for those in the heavy smoking category. This result would seem to find support in the observations of Hueper (1957), that the lung cancer rate in Austria was about twice as high in communities located on main traffic arteries than in those situated remote from main highways, and Kretz (1953) who stated that, in Vienna, among the three most frequent sites of cancer, the lung takes first place for the following occupations: Traffic (9·7 per cent), Iron and Metal industry (8·7 per cent), Building (5·1 per cent), Hotel and Licensed Bar-keeping (3·6 per cent) and several branches of industry (17·2 per cent).

It is well known that during recent decades the rise in lung cancer frequency is paralleled by a similar rise in consumption of motor fuel. Benzene extracts of vehicular exhausts have been shown by Kotin, Falk and Thomas (1954, 1955) to produce skin cancers in mice.

As a means towards assessing the relative importance of vehicular exhaust-polluted atmospheres vis-a-vis cigarette smoking in the aetiology of lung cancer, at laboratory level, it would be desirable, firstly, to gauge the carcinogenic potency of the atmospheric extracts and cigarette smoke condensates in terms of, say, benzopyrene units of activity. Secondly, the necessity arises of comparing and contrasting the spectra of carcinogens in both forms of air pollution, the general and the individualised, with the view to furnishing a rational basis for the epidemiological results. The present paper, which records the identification of further carcinogens and other compounds in vehicular exhausts, contributes to the latter point and is an extension of previously reported work (Lyons and Johnston, 1957).

### EXPERIMENTAL

Alternate adsorption chromatography on alumina and silica gel was carried out to purify fractions, isolated in the previous investigation from diesel and petrol engine exhaust soots. A number of compounds had since become available for reference. Identification of unknown compounds was made by comparing their ultra-violet absorption and fluorescence spectra (excitation radiation, 365 m$\mu$.) with the spectra of reference compounds.

By these methods the following compounds have been newly identified in eluates succeeding 3,4-benzopyrene in petrol exhaust chromatograms: 3,4-benzofluoranthene, tetracene (naphthacene), pentaphene, 1,2,3,4-dibenzopyrene, 11,12-benzofluoranthene, 1,2,9,10-dibenzotetracene, 1,2,4,5-dibenzopyrene and 1,12,2,3-dibenzoperylene.

In the diesel exhausts, the following compounds were similarly identified: 3,4-benzofluoranthene, pentaphene, 1,2,3,4-dibenzopyrene, 11,12-benzofluoranthene, 1,2,9,10-dibenzotetracene.

Of the above listed compounds, the 3,4-benzofluoranthene, 1,2,3,4-dibenzopyrene and 1,2,4,5-dibenzopyrene are carcinogenic, while the 1,12,2,3-dibenzoperylene, it is believed, has not yet been tested. The structural formulae of these four compounds are shown in Fig. 1.

I

II

III

IV

FIG. 1.—Structural formulae of compounds identified in vehicular exhausts.
I 3,4-Benzofluoranthene. II 1,2,3,4-Dibenzopyrene.
III 1,2,4,5-Dibenzopyrene. IV 1,12,2,3-Dibenzoperylene.

3,4-Benzofluoranthene has an intense blue fluorescence (the 11,12-derivative had an intense blue-violet fluorescence) and closely follows 3,4-benzopyrene on the chromatogram. The absorption spectrum of the compound from the petrol exhaust soot, which showed maxima at 368, 351, 303, 294, 275, and 256 m$\mu$. in cyclohexane, is presented (Fig. 2). The fluorescence spectrum, which did not reveal any striking or discrete bands, consisted of a region of absorption commencing at 295 m$\mu$. and having two peaks at 428 and 450 m$\mu$.

FIG. 2.—Ultraviolet absorption spectra : (solvent : cyclohexane).
——— 3,4-Benzofluoranthene from petrol exhaust soot.
– – – – Standard 3,4-Benzofluoranthene.

The absorption spectrum in benzene of 1,2,3,4-dibenzopyrene from petrol soot is shown in Fig. 3. The compound is associated with a green fluorescence.

FIG. 3.—Ultraviolet absorption spectra : (solvent : benzene).
——— 1,2,3,4-Dibenzopyrene from petrol exhaust soot.
– – – – Standard 1,2,3,4-Dibenzopyrene.

The 1,2,4,5-dibenzopyrene eluates from petrol soot had a blue fluorescence. The absorption spectrum, in benzene (Fig. 4), gave peaks at 428, 416, 395, 378, 360, 327, 306, and 296 mμ., while fluorescence maxima at 416, and 440 mμ. were obtained.

Eluates containing the 1,12,2,3-dibenzoperylene had a blue-violet fluorescence. The absorption spectrum (Fig. 5) using benzene as solvent, showed maxima at 405, 391, 377, 357, 344, 309, and 297 mμ. A peak at 421·5 mμ. given by Clar

(1952) for this compound is now believed to be due to an impurity (Clar, personal communication). The fluorescence spectrum is distinctive and possesses a series

FIG. 4.—Ultraviolet absoprtion spectra : (solvent : benzene).
——— 1,2,4,5-Dibenzopyrene from petrol exhaust soot.
– – – – Standard 1,2,4,5-Dibenzopyrene.

FIG. 5.—Ultraviolet absorption spectra : (solvent : benzene).
——— 1,12,2,3-Dibenzoperylene from petrol exhaust soot.
– – – – Standard 1,12,2,3-Dibenzoperylene.

of sharp bands at 404, 418, 428, 443 and 453 mμ. This spectrum was previously shown (Lyons and Johnston, 1957, Fluorescence Spectrum XVII) superimposed upon another band system which had maxima at 450, 467, and 478 mμ. Absorption maxima, in benzene, for the latter still unidentified compound, following chromatographic separation from the dibenzoperylene were obtained at 422, 396, 369, 347, 328 and 304 mμ. The absorption spectrum of the incompletely purified compound was shown in Fig. 5 of the previous paper, while that of the then unidentified dibenzoperylene was shown in Fig. 4.

The fluorescence spectra of the benzofluoranthene, the two dibenzopyrenes and the dibenzoperylene are shown in Fig. 6. It has not yet been possible to estimate the 3,4-benzofluoranthene or the 1,12,2,3-dibenzoperylene. 1,2,3,4-Dibenzopyrene was found to occur at a concentration of approximately 22 parts

FIG. 6.—Fluorescence spectra of compounds derived from petrol exhaust soot: (excitation at 365 mμ).

I 3,4-Benzofluoranthene; II 1,2,3,4-Dibenzopyrene; III 1,2,4,5-Dibenzopyrene; IV 1,12,2,3-Dibenzoperylene.

per million of petrol soot and 14 parts per million of diesel soot. The 1,2,4,5-dibenzopyrene occurred in the petrol soot at a concentration of about 10 parts per million.

### DISCUSSION

3,4-Benzofluoranthene, tetracene, 1,2,3,4-dibenzopyrene, pentaphene and 1,2,9,10-dibenzotetracene, here recorded as occurring in vehicular exhausts, have been reported by Wynder and Wright (1957) as present in cigarette smoke condensates. The present author (1958) has also detected and estimated 1,2,3,4-dibenzopyrene in cigarette smoke.

3,4-Benzofluoranthene is the most recent hydrocarbon and the first fluoranthene derivative found to be carcinogenic. According to preliminary results obtained for mouse skin by Wynder in New York (Wynder, personal communication)

the compound is not much below 3,4-benzopyrene in carcinogenic potency, producing tumours in 100 per cent of the animals at 0·5 per cent concentration and even at 0·1 per cent is expected to produce lesions in the majority of animals.

1,2,4,5-Dibenzopyrene shares the property exhibited by the 1,2,3,4-, 3,4,8,9- and 3,4,9,10-derivatives of being a potent carcinogen (Arbuzov and Grechkin, 1952, 1953). In so far as is known, this is the first occasion on which the presence of 1,2,4,5-dibenzopyrene and 1,12,2,3-dibenzoperylene in any pyrogenic material has been shown.

### SUMMARY

Fractions isolated from petrol engine and diesel engine exhaust samples were assayed for the presence of polycyclic aromatic hydrocarbons. The fractions examined had all succeeded 3,4-benzopyrene in initial chromatographic runs.

The methods employed were repetitive adsorption chromatography on alumina and silica gel, followed by ultra-violet absorption and spectrographic analysis of the eluates. By these methods the following compounds were identified in the petrol engine exhaust sample : 3,4-benzofluoranthene, tetracene (naphthacene), pentaphene, 1,2,3,4-dibenzopyrene, 11, 12-benzofluoranthene, 1,2,9,10-dibenzotetracene, 1,2,4,5-dibenzopyrene, and 1,12,2,3-dibenzoperylene. In the diesel engine exhaust sample, the following compounds were identified : 3,4-benzofluoranthene, pentaphene, 1,2,3,4-dibenzopyrene, 11,12-benzofluoranthene and 1,2,9,10-dibenzotetracene.

Of the above listed compounds, the 3,4-benzofluoranthene, 1,2,3,4-dibenzopyrene and 1,2,4,5-dibenzopyrene are carcinogenic, while the 1,12,2,3-dibenzoperylene, it is believed, has not yet been tested. This is the first time, it is thought, that the latter two compounds have been identified in any pyrogenic material.

I wish to thank Dr. P. R. Peacock, Director of Research, for helpful advice, and Dr. E. Clar, Chemistry Department, University of Glasgow, for the gift of samples of polycyclic aromatic hydrocarbons.

### REFERENCES

ARBUZOV, B. A. AND GRECHKIN, N. P.—(1952) *Zh. Obshch. Khim.*, **22**, 1692.—(1953) *Chem. Abstr.*, **47**, 9953 h.

CLAR, E.—(1952) ' Aromatische Kohlenwasserstoffe '. Berlin (Springer).

HAENSZEL, W. AND SCHIMKIN, M. B.—(1956) *J. nat. Cancer Inst.*, **16**, 1417.

HAMMOND, E. C. AND HORN, D.—(1958) *J. Amer. med. Ass.*, **166**, 1294.

HUEPER, W. E.—(1957) ' Cancer ', Vol. 1, p. 46. Ed. R. W. Raven, London (Butterworth).

KOTIN, P., FALK, H. L. AND THOMAS, M.—(1954) *Arch. industr. Hyg.*, **9**, 164.—(1955) *Ibid.*, **11**, 113.

KRETZ, J.—(1953) *Acta Un. int. Cancr.*, **9**, 542.

LYONS, M. J.—(1958) *Nature, Lond.*, **182**, 178.

*Idem* AND JOHNSTON, H.—(1957) *Brit. J. Cancer*, **11**, 60.

MILLS, C. A. AND PORTER, M. M.—(1957) *Cancer Res.*, **17**, 981.

STOCKS, P.—(1957) *Rep. Brit. Emp. Cancer Campaign*, Supplement to Part II.

WYNDER, E. L. AND WRIGHT, G.—(1957) *Cancer*, **10**, 255.